# Supplementary material for: Evidence supported by Mendelian randomization: impact on inflammatory factors in knee osteoarthritis
Source: Front Med (Lausanne). 2024 May 28;11:1382836. doi: 10.3389/fmed.2024.1382836 (PMC11165061; doi:10.3389/fmed.2024.1382836)
Supplement: Supplementary file 1 [file Data_Sheet_1.ZIP › data/GCST90274790_5e6/again/790.forest.pdf]

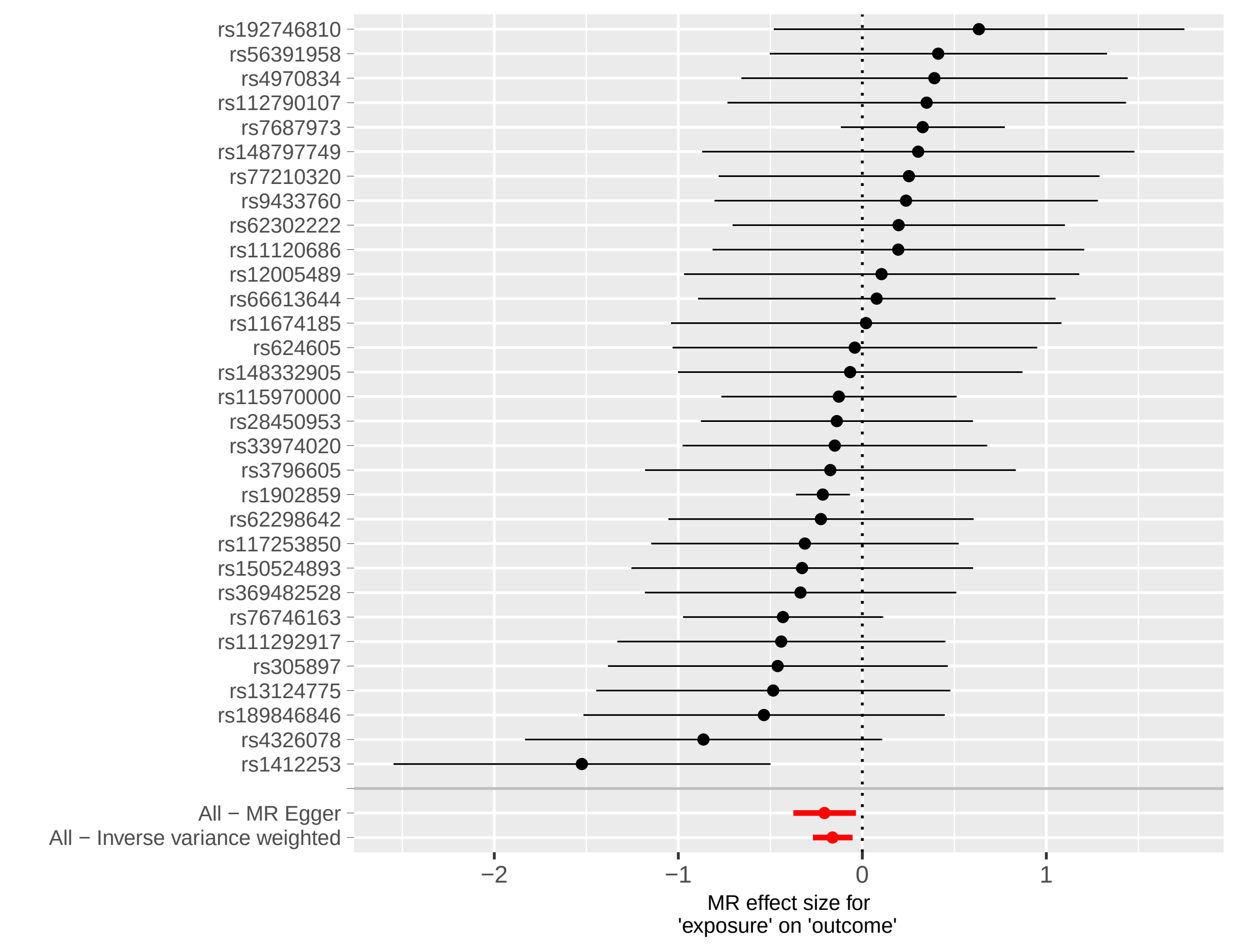
rs192746810
rs56391958
rs4970834
rs112790107
rs7687973
rs148797749
rs77210320
rs9433760
rs62302222
rs11120686
rs12005489
rs66613644
rs11674185
rs624605
rs148332905
rs115970000
rs28450953
rs33974020
rs3796605
rs1902859
rs62298642
rs117253850
rs150524893
rs369482528
rs76746163
rs111292917
rs305897
rs13124775
rs189846846
rs4326078
rs1412253
All − MR Egger
All − Inverse variance weighted
−2
−1
0
1
MR effect size for
'exposure' on 'outcome'
